# A scoping review of UK local government workplace health and wellbeing programmes

**DOI:** 10.1186/s12889-025-23176-3

**Published:** 2025-06-07

**Authors:** Austen El-Osta, Sami Altalib, Aos Alaa, Mahmoud Al-Ammouri, Manisha Karki, Eva Riboli-Sasco, Azeem Majeed, Laura Kudrna

**Affiliations:** 1 Self-Care Academic Research Unit (SCARU), School of Public Health, Imperial College London, 90 Wood Lane, London, W12 0BZ UK; 2 Department of Primary Care & Public Health, School of Public Health, Imperial College London, 90 Wood Lane, London, W12 0BZ UK; 3 Institute of Applied Health Research in Birmingham, Birmingham, B15 2 TT UK

**Keywords:** Employee health, Public health, Sickness absence, UK, Wellbeing initiatives, Workplace health

## Abstract

**Background:**

Workplace settings are linked to staff health and wellbeing, affecting sickness absence, presenteeism and productivity. With the growing prevalence of health issues among employees in the UK, including stress and long-term conditions, effective workplace health and wellbeing support by local government can play a crucial role in keeping people economically active and well.

**Objective:**

Identify and characterise workplace health and wellbeing programmes offered by local authorities within the United Kingdom.

**Methods:**

A scoping review involved a comprehensive search of Local Authority Districts (LADs) and county councils' websites followed by direct communications between 1 January 2024 and 30 April 2024. Initiatives were included if they were designed to enhance workplace health and wellbeing, actively ongoing and offered at no cost to workplaces. Data were extracted on the initiative name, provider, deprivation level, health focus, workplace eligibility and accreditation processes.

**Results:**

The review identified 61 active local government workplace health programmes across the UK in March 2024, reflecting a 21% provision among local authorities. These initiatives were homogenous in focus, scope of coverage and implementation methods, with all focusing on general health. Geographical mapping highlighted regional disparities in the provision of workplace health and wellbeing initiatives that are free at the point of access (WHISPAs). England had a higher number (Central, Southern, and Southeastern regions specifically) while the rest of England, Wales, Scotland and North Ireland had a lower number or no WHISPAs.

**Conclusion:**

There is a need for more coordinated efforts to increase the visibility and accessibility of local government workplace health initiatives that are free at the point of access. National workplace health accreditation could further encourage employers to adopt health and wellbeing programmes.

## Introduction

The health and wellbeing of the UK workforce have become an increasingly pressing concern in recent years and especially since the advent of the COVID-19 pandemic. The pandemic also accelerated changes in work practices, including the widespread adoption of remote and flexible working, which have introduced new challenges and opportunities for supporting employee wellbeing [1, 2]. These developments have prompted both national and local governments to prioritise workplace health as a critical component of the recovery agenda, with renewed focus on the role of local authorities in supporting employers and employees to navigate ongoing risks and adapt to evolving workplace environments [3].

In 2022, the sickness absence rate in the UK reached 2.6%, the highest level since 2004, resulting in an estimated 185.6 million working days lost. The predominant reasons for these absences were minor illnesses, musculoskeletal (MSK) problems and mental health conditions, including stress, anxiety and depression [4]. This growing burden of workplace ill-health is estimated to cost the UK economy over £100 billion annually, which includes costs related to sickness absence, reduced productivity due to unemployment, the provision of informal care and productivity losses [5]. Working and workplaces matter because they affect substantial numbers of people. It is estimated that more than 33 million individuals aged 16 and over are employed in the UK [6]. Generally, individuals spend nearly one-third of their lifetime at work, whereas in the UK the average weekly working hours of full-time employees is 36.7 h [7].

For these reasons, workplace environments play a crucial role in shaping employee health and wellbeing and could directly influence the extent to which individuals can self-care in this setting [8]. Earlier studies consistently highlighted the significance of the workplace as a key setting for promoting holistic health and preventing long-term conditions [9, 10]. However, the concept of ‘work as a health outcome’ has historically received less research attention than other domains such as community-based or clinical interventions. Recent reviews note that despite the well-established impact of work on health and the development of effective occupational health interventions, there remain substantial gaps in the evidence base-particularly regarding the reach, adoption, and long-term effectiveness of workplace health promotion programmes [11]. Moreover, employers often fail to capitalise on this opportunity, with research indicating that many organisations do not implement effective workplace health and wellbeing initiatives [12–14]. Employers may be motivated to offer initiatives for various reasons, including boosting productivity, reducing economic setbacks and improving staff retention [15–17]. While workplace health programmes can have a positive impact on both organisational and individual health, there are many barriers to their adoption and effectiveness.

A systematic review has highlighted several significant obstacles to the adoption and effectiveness of workplace health and mental wellbeing practices [18]. These include organisational barriers like insufficient support from management, inadequate resources, and a lack of integration into the organisational culture. Without the commitment of senior management, these initiatives often lack the resources and strategic prioritisation needed for success [8]. Limited financial resources, time constraints, and a shortage of personnel further impede the long-term implementation of wellbeing practices which may be seen as secondary to core business operations. Additionally, practices that are not seamlessly integrated into daily routines may be perceived as peripheral rather than integral, diminishing their perceived importance and efficacy [19, 20]. Individual barriers, such as employee resistance due to a lack of understanding, scepticism about benefits, or fear of stigma, can also hinder engagement. High workloads and job stress can further reduce employees'ability to participate in wellbeing programmes [18, 21–23]. Contextual barriers, like external economic pressures, often cause organisations to prioritise productivity and cost-cutting over employee wellbeing. Industry-specific challenges, such as shift work and hazardous conditions, present unique implementation obstacles. Moreover, program-specific barriers like one-size-fits-all approaches and a lack of evidence-based interventions can compromise effectiveness [24, 25]. Addressing these barriers through strategic planning, resource allocation, and employee engagement is crucial for enhancing the adoption and effectiveness of workplace health and psychological wellbeing initiatives [26].

In addition to organisational and individual barriers, broader contextual factors can play a critical role in shaping workplace health and wellbeing. For example, some business models are predicated on maximising labour flexibility or reducing costs, sometimes resulting in poor-quality jobs, high work intensity, or job insecurity-all of which are linked to adverse health outcomes and reduced effectiveness of wellbeing interventions [27]. Features of the UK’s liberal welfare regime such as welfare conditionality, may also compel people with health conditions to pursue or remain in unsuitable work, which can exacerbate health inequalities and negatively affect wellbeing [28].

One significant barrier of the widescale adoption of workplace health initiatives is the perceived high initial costs [29]. Additionally, a lack of awareness about workplace health efforts in other companies may lead employers to believe that not offering such initiatives is the norm [30]. Descriptive social norms are shaped by observing the actions of others and influence perceptions of how widespread and feasible certain behaviours are. These norms could be altered by making workplace health initiatives more visible. This increased visibility could encourage more employers to adopt and implement them [31, 32]. National workplace health accreditation schemes are valuable tools for promoting, evaluating, and acknowledging the existence and feasibility of such initiatives. The evaluation also revealed low levels of both employer provision and employee participation in these initiatives. Employers emphasised that successful programmes depend on management support, effective communication, the ability to tailor programmes to meet local needs, and acknowledgement of accomplishments [1].

The transition to remote work since the advent of the COVID-19 pandemic has altered the factors contributing to mental and physical health challenges, including anxiety, depression, MSK issues and other conditions. These effects may vary across different demographic groups, highlighting the crucial need for a strengthened focus on workplace and occupational health measures [2].

Workplaces offer opportunities for health promotion, but adverse conditions can damage physical and mental health. Psychosocial stressors including high demands, low autonomy and poor support are linked to cardiovascular disease, depression, anxiety and MSK disorders [33]. The Whitehall II study showed that chronic work stress and low control predict coronary heart disease and metabolic syndrome via behavioural and neuroendocrine pathways [34]. Precarious employment, long hours and effort-reward imbalance further increase risks of anxiety, burnout and mortality, especially among lower socioeconomic groups and migrants [35, 36]. In the UK, occupational factors cause 23% of work-related ill health, with stress, depression, or anxiety accounting for 49% of cases and 17.1 million lost working days in 2022/23 [37].These risks reinforce health inequalities among lower-income and high-strain workers.

Local Authorities (LAs) in the UK are major employers & statutory public health leaders. They implement national health priorities & convene cross-sector partnerships to extend workplace health initiatives to local businesses and Small and medium size enterprises (SMEs). Given persistent health inequalities & the role of work in shaping health outcomes, LA actions are crucial for improving population health. Understanding current LA workplace health & wellbeing programmes is essential to identify gaps, inform policy and support more equitable, effective interventions [38, 39].

There are significant barriers to the adoption and implementation of workplace health initiatives, including costs and varying local contexts that shape the need for enhanced workplace health promotion and prevention strategies. One approach to overcoming these barriers is offering free health initiatives within local governments, although their provision remains inconsistent. Previous research indicates that incentivising companies to adopt local government health initiatives can stimulate progress [40]. Monetary incentives, such as partial reimbursement of programme costs, have been shown to increase SME participation in workplace health initiatives, especially when paired with advisory support [40]. Non-monetary incentives like accreditation also encourage adoption by signalling credibility and peer uptake [20]. However, while incentives boost initial participation, evidence is mixed regarding their impact on the sustained implementation and long-term health outcomes, particularly for smaller firms with limited resources.

This study is informed by the wellbeing policy-making framework [41], which recognises that local government has a central role in maximising population wellbeing by addressing the social, economic and environmental determinants of health. Health and Wellbeing Strategies required of local authorities in England exemplify this approach by integrating evidence of need with interventions that target key drivers of health, including work and employment [42]. Work is widely recognised as a critical determinant of health, and local authorities are uniquely positioned to influence both their own workforce and those of local employers [43]. Despite this, provision of workplace health and wellbeing initiatives remains fragmented and unevenly distributed, with limited evidence on the scope and characteristics of programmes that are free at the point of access and led by local authorities [3].

Given the rising rates of work-related ill health, persistent health inequalities and recent policy momentum to prioritise workplace health at both local and national levels, the aim of this study was to identify and characterise local government workplace health and wellbeing initiatives across the UK. A second objective was to highlight the distribution of these health and wellbeing initiatives that are free at the point of access to employers across the UK.

## Methods

The reporting of methods follows the PRISMA-ScR headings.

### Protocol and registration

The protocol is available on the Open Science Foundation [44] and can be accessed by following this link: https://osf.io/mfpvx/

### Eligibility criteria

The inclusion and exclusion criteria are shown in Table 1. We included both published and unpublished local government workplace health programmes. They were documented in English and provided in the UK primarily by Local Authority Districts (LADs) councils and/or county councils. They needed to be for workplaces in the local community (rather than only local government staff). Eligible initiatives also needed to be free at the point of use to workplaces and designed specifically to enhance health and wellbeing within workplace environments. To ensure the review reflects contemporary practices and trends, active initiatives during the scoping review (from 1 January 2024 and 30 April 2024) were included. We excluded initiatives that were no longer active, in the planning stages (i.e. yet to launch), focussed entirely on occupational health and safety (versus staff health and wellbeing more generally) and those only for local government staff rather than the local workplace community.

**Table 1:** Inclusion and exclusion criteria

Inclusion criteria	Exclusion criteria
• Published online or unpublished • Initiatives must be documented in English • Provided within UK (England, Scotland, Wales and Northern Ireland) • Offered by LADs and/or county councils • For local workplace community • Accessible free of charge to employers and their employees • Designed to enhance workplace health and wellbeing • Must be active in March 2024	• Initiatives are in the planning stages or have yet to be launched • Initiatives that are no longer active • Initiatives focuses entirely on occupational health and safety rather than staff health and wellbeing more generally

### Information sources

To identify relevant local government workplace health offerings, we conducted a multi-stage process involving both web-based and direct engagement approaches. From January to April 2024, we systematically reviewed official websites, reports and documentation available online using a series of targeted searches on Google for all 382 UK local authorities, including both lower-tier and upper-tier councils. For councils where no identifiable workplace health offering was found online, we initiated direct inquiries via email to the relevant local authority or county council between March and May 2024. If no response was received after three weeks, we followed up with additional emails and, where necessary, submitted a Freedom of Information (FOI) requests under the Freedom of Information Act 2000. All communications with LAs were completed by May 2024. This direct engagement was essential, as information about the availability, cost and eligibility of workplace health programmes is not always clearly stated online.

Local government structures in the UK vary by nation and region. In England, many areas operate a two-tier system, with upper-tier county councils responsible for services such as education, transport, and social care, and lower-tier district councils providing more localised services like housing and waste collection. Some areas, including most large cities and urban regions, use single-tier unitary authorities that deliver all local government functions. In contrast, Scotland, Wales, and Northern Ireland operate exclusively with single-tier councils (unitary authorities), although the range of powers and responsibilities may differ between nations. A list of lower-tier and upper-tier local authorities in England with their respective index of multiple deprivation (IMD) score and rank (2019) was retrieved from the Ministry of Housing, Communities and Local Governments’ publicly available data [45] and from government websites in the UK, Scotland, Wales and Northern Ireland [46–49].

### Search

The search strategy was designed to comprehensively capture the spectrum of local government workplace health programmes on LAD and county councils'official websites. A strategic combination of specific keywords was used, and the search strings were constructed to include terms directly related to workplace health initiatives.

The final list of search terms used was:'workplace health';'workplace wellbeing';'workplace health award';'workplace health initiative';'employees’ health';'employee wellbeing'. Because the specific status of initiatives-such as being “free at the point of access” or “available to the community”, was rarely indicated in programme titles or online descriptions, it was not practical to include these as search terms. Therefore, we adopted a two-stage approach: first, broad search terms were used to identify all potentially relevant workplace health initiatives; second, inclusion and exclusion criteria regarding cost and accessibility were applied during the screening and verification process.

Information about local government workplace health programmes was also supplemented with integrated knowledge transition [50], including perspectives from a large professional network of stakeholders who are members of the WHISPA workplace health and wellbeing network (www.whispas.co.uk) and Directors of Public Health across the UK. Councils without accessible WHISPA information online were contacted directly and followed up. WHISPAs identified in England, Scotland, Northern Ireland, and Wales were screened for eligibility using our predefined list of criteria; only those meeting the inclusion criteria were included. Where no response from the LA was received after three weeks, it was assumed that this information was not forthcoming. When required, a FOI was completed to engage with the LA.

### Selection of sources of evidence

Screening and eligibility assessments were conducted using a multi-step process. First, all reviewers (MA, MK, AA and ERS) searched the internet to identify each council's official website and contact details. A shared Excel sheet was created and completed to capture relevant information from each LA. Second, two reviewers (MA, MK, AA, and/or ERS) reviewed the council's official website and used the search terms to explore the availability of local government workplace health provision in that region. Two reviewers were used to increase the consistency of selection. All relevant and potential initiatives were examined against the inclusion and exclusion criteria. In cases of disagreement between the initial reviewers, a third reviewer (AA or SA) resolved the conflict. In all instances, a complete agreement was reached. All eligible initiatives were added to the Excel sheet along with their URL link.

### Data charting process

To ensure a consistent and systematic approach to charting, a bespoke template was created by all co-authors. Data charting was carried out independently by SA, AA and MA, with periodic meetings to compare results and resolve discrepancies iteratively, ensuring consistency.

### Data items

The charted variables were the councils'IMD score and rank, the name of the local government workplace health offering, administering LAD or county council, cost-free accessibility, contact email and website link. Additional characterisation involved identifying the owner/provider, provider type, year of establishment, area of coverage, employers eligible to benefit from the initiative, primary health focus, type of scheme, accreditation process/framework and levels of accreditation. Deprivation levels for LADs in England were determined using the English Indices of Deprivation 2019, published by the Ministry of Housing, Communities and Local Government. The average score for each LAD, which aggregates deprivation across seven domains (income, employment, education, health, crime, housing, and environment), was extracted from the publicly available dataset [51]. These scores were ranked by decile decile, with the first decile representing the most deprived 10% of LADs nationally. Scotland, Wales and Northern Ireland publish their own deprivation indices, which are constructed using different domains, weights and spatial units, making direct comparison across nations problematic. Recent research has demonstrated that naïvely combining or comparing country-specific deprivation scores can lead to misleading results in UK-wide analyses, as deprivation quintiles or deciles are not equivalent between countries. To avoid introducing bias or spurious comparisons, deprivation scores and deciles were not calculated or reported for local authorities in Wales, Scotland, or Northern Ireland [52].

The data extraction tool included a column to capture information about any accreditation if offered by a WHISPA. The accreditation schemes set specific criteria for each level and formally assess and recognise participating employers [53]. Not all WHISPAs offered accreditation levels (e.g., bronze, silver, gold) that are typically awarded by local authority-led healthy workplace accreditation schemes with support from Public Health England, the Local Government Association, and the Association of Directors of Public Health.

### Critical appraisal of individual sources of evidence

In keeping with guidance on scoping reviews [54], a critical appraisal of evidence was not conducted. Our study focused on cataloguing existing offerings rather than assessing their efficacy to create a comprehensive baseline of available programmes for future evaluative research, which could then focus on the quality and effectiveness of the initiatives.

### Synthesis of results

The synthesis of charted data involved aggregating and summarising information to provide a detailed overview of offerings across the UK. These categories included the geographical distribution, administration methods, provider types, years established, coverage areas, eligibility criteria for workplaces and the primary health focus(es) of the initiatives. By categorising the data, we were able to identify patterns and variations in the provision of workplace health and wellbeing programmes, as well as highlight disparities and gaps in service coverage that can guide future enhancements and targeted interventions. Descriptive statistics were used to analyse the prevalence and characteristics of the WHISPAs. Results were visually presented using a geographical map to illustrate patterns and trends across different regions. Vector data representing administration boundaries was obtained from the Office for National Statistics (ONS), which is publicly available to map the LADs and counties [55]. The publicly available Free and Open-Source Software (FOSS) QGIS was used to create the maps and present the data [56]. Additionally, a characterisation table was created to display the list of LADs and county councils offering workplace support, their level of deprivation using IMD score and rank and the names and key attributes of each WHISPA. These allowed for a clear depiction of the current landscape of workplace health initiatives, which might help inform easy interpretation and identification of gaps in provision.

## Results

### Selection of sources of evidence

**Fig. 1:**
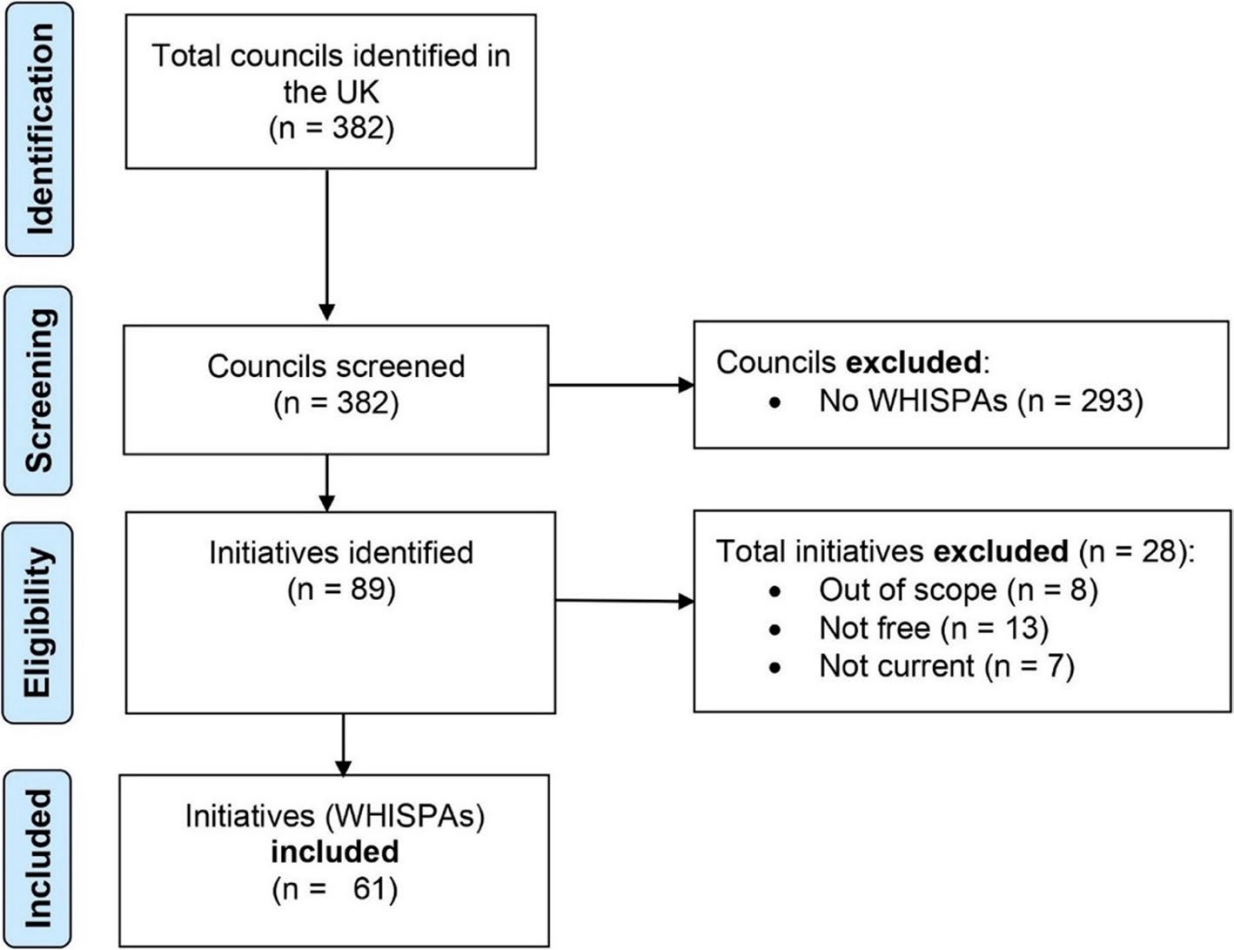
PRISMA flowchart showing the process of identifying councils and inclusion of workplace and wellbeing initiatives

There were 382 councils identified in the UK (317 in England, 32 in Scotland, 22 in Wales, and 11 in Northern Ireland). After screening, 293 councils were excluded, leaving 89 councils. From this subset, 28 were excluded based on eligibility (8 were out of scope, 13 were not free at the point of use, and 7 were not current). This resulted in a total of 61 councils with a local government workplace offering. Figure 1 provides the PRIMSA flow diagram detailing the selection process.

### Characteristics and results the sources of evidence

A total of 38 FOIs were processed by different LAs and responses were received according to the Freedom of Information Act 2000 [57]. Key information about each local authority WHISPA is presented in Table 2, including the council’s name, IMD score, provider type, year, coverage, workplace eligibility, primary health focus, scheme type and accreditation.

**Table 2:** Characteristics of workplace health and wellbeing initiatives that are free at the point of use to workplaces (WHISPAs)

Council	Administration	IMD score (Decile)	WHISPA	Citation	Owner/Provider	Provider Type	Year est	Coverage	Eligible Org	Primary Health Focus	Scheme Type	Accreditation level
1. Adur District Council	England – non-metropolitan district	17.594 (6)	Adur & Worthing Wellbeing (Workplace Health)	[58]	West Sussex County Council & Adur & Worthing Council	Local government	NA	Local	No restrictions	General health	one-to-one telephone or virtual appointments, talks & workshops	Not specified
2. Arun District Council	England – non-metropolitan district	18.638 (5)	Arun Wellbeing (Workplace Health)	[59]	West Sussex County Council & Arun District Council	Local government	NA	Local	No restrictions	General health	one-to-one telephone or virtual appointments, talks & workshops	Not specified
3. Cheltenham Borough Council	England – non-metropolitan district	14.26 (7)	The Healthy Workplaces Gloucestershire	[60]	Gloucestershire County Council	County government	2019	Regional	No restrictions	General health	Assessments, provision of resources & support to improve workplace health	Foundation & Enhanced levels
4. Chichester District Council	England – non-metropolitan district	14.085 (8)	Chichester Wellbeing (Workplace Health)	[61]	West Sussex County Council & Chichester District Council	Local government	NA	Local	No restrictions	Geneal health	one-to-one telephone or virtual appointments, talks & workshops	Not specified
5. Crawley Borough Council	England – non-metropolitan district	18.94 (5)	Crawley Wellbeing (Workplace Health)	[62]	West Sussex County Council & Crawley Borough Council	Local government	NA	Local	No restrictions	General health	One-to-one appointments, Talks, presentations & Information stands	Not specified
6. East Hertfordshire District Council	England – non-metropolitan district	18.890 (5)	Hertfordshire Healthy Workplace	[63]	Hertfordshire County Council	County government	2014	Regional	SMEs from the private & voluntary sector	General health	Provision of resources & training & support to develop workplace health champions	Level 1 (Foundation), level 2 (workplace health holistic view) & level 3 (local community health promotion)
7. East Staffordshire Borough Council	England – non-metropolitan district	8.188 (10)	Workplace Health Staffordshire	[64]	Staffordshire County Council	County government	NA	Regional	No restrictions	General health	Awards programme & resources	Not specified
8. Horsham District Council	England – non-metropolitan district	9.89 (10)	Horsham District Wellbeing (Workplace Health)	[65]	West Sussex County Council & Horsham District Council	Local government	NA	Local	No restrictions	General health	one-to-one telephone or virtual appointments, talks & workshops	Not specified
9. Maldon District Council	England – non-metropolitan district	14.169 (8)	Working Well in Essex	[66]	Essex County Council & Maldon District Council	County government	NA	Regional	No restrictions	General health	Meetings, resources, trainings, development programme & e-newsletter	Level 1 (five actions), level 2 (additional six actions) & level 3 (further 10 actions)
10. Mid Sussex District Council	England – non-metropolitan district	7.747 (10)	Mid Sussex Wellbeing (Workplace Health)	[67]	West Sussex County Council & Mid Sussex District Council	Local government	2012	Local	No restrictions	General health	one-to-one telephone or virtual appointments, talks & workshops	Not specified
11. Wealden District Council	England – non-metropolitan district	12.311 (9)	East Sussex Wellbeing at Work Programme	[68]	East Sussex County Council	County government	2021	Regional	No restrictions	General health	Resources, training, events & a free Accreditation Scheme	Bronze, Silver & Gold
12. West Lancashire District Council	England – non-metropolitan district	18.645 (5)	Workplace Wellbeing (The NEST)	[69]	West Lancashire District Council	Local government	2024	Local	No restrictions	General health	Guidance, trainings, courses & frameworks	Not specified
13. Worthing Borough Council	England – non-metropolitan district	17.012 (6)	Adur & Worthing Wellbeing (Workplace Health)	[58]	West Sussex County Council & Adur & Worthing Council	Local government	NA	Local	No restrictions	General health	one-to-one telephone or virtual appointments, talks & workshops	Not specified
14. Bedford Borough Council	England – unitary authorities	18.932 (5)	Workplace Health & Wellbeing Service Offer	[70]	Bedford Borough Council, Central Bedfordshire & Milton Keynes City Council	Local government	2022	Regional	No restrictions	General health	Free training & workshops, support for adopting healthy lifestyles & framework	Not specified
15. Blackburn with Darwen Borough Council	England – unitary authorities	36.013 (1)	Business Health Matters	[71]	Blackburn with Darwen Borough Council & Business Health Matters	Local government	NA	Local	No restrictions	General health	Strategy that comprises of physical & mental health support	Explorer, adventurer & trailblazer
16. Blackpool Council	England – unitary authorities	45.039 (1)	Healthier Workplaces	[72]	Blackpool Council	Local government	NA	Local	No restrictions	General health focusing on mental health	support & resources	Not specified
17. Bracknell Forest Borough Council	England – unitary authorities	10.241 (10)	Healthy Workplace Alliance	[73]	Bracknell Forest Borough Council	Local Government	NA	Local	No restrictions	General health including mental health & MSK conditions	Meetings for sharing best practices, a forum for communication, access to local services & support & a local Healthy Workplace Award for accreditation	Not specified
18. Brighton & Hove City Council	England – unitary authorities	20.761 (4)	Workplace Health	[74]	Brighton & Hove City Council	Local government	NA	Local	No restriction	General health	Support for developing workplace wellbeing programmes, health promotion campaigns, health & wellbeing resources & health checks	Not specified
19. Central Bedfordshire Council	England – unitary authorities	12.152 (9)	Workplace Health & Wellbeing Service Offer	[75]	Bedford Borough Council, Central Bedfordshire & Milton Keynes City Council	Local government	2022	Regional	No restrictions	General health	Offers free health & wellbeing services, resources, training & support for healthy lifestyle changes	Not specified
20. Cornwall Council	England – unitary authorities	23.072 (3)	Healthy Cornwall Award	[76]	Cornwall Council	Local government	NA	Local	No restrictions	General health	Health support, training services & award	Not specified
21. Durham County Council	England – unitary authorities	26.793 (2)	Health & wellbeing at work	[77]	Durham County Council	Local government	2015	Regional	No restrictions	General health focusing on mental health	Training hub & award	Bronze, Silver, Gold, Continuing Excellence & Maintaining Excellence
22. Hartlepool Borough Council	England – unitary authorities	35.037 (1)	Better Health at Work Award (BHAWA)	[78]	Hartlepool Borough Council & BHAWA programme	Local government	2009	Local	No restrictions	General health	Increased access to health information & interventions	Bronze, Silver, Gold, Continuing Excellence, Continuing Excellence Plus & Maintaining Excellence
23. Leicester City Council	England – unitary authorities	30.877 (1)	Our Healthy Workplace	[79]	Leicester City Council	Local government	NA	Local	No restrictions	General health	Focus groups & strategic planning sessions to engage employees in the development of health & wellbeing action plans	Not specified
24. Middlesbrough Borough Council	England – unitary authorities	40.460 (1)	Better Health at Work Award (BHAWA)	[80]	Middlesbrough Borough Council	Local Government	2009	Local	No restrictions	General health	Increased access to health information & interventions	Bronze, Silver, Gold, Continuing Excellence, Continuing Excellence Plus & Maintaining Excellence
25. Milton Keynes Council	England – unitary authorities	17.980 (6)	The Healthy Workplace Standards/Healthy Workplace Award	[81]	Bedford Borough Council, Central Bedfordshire & Milton Keynes City Council	Local government	2022	Local	No restrictions	General health	Free training & workshops, support for adopting healthy lifestyles & framework	Not specified
26. North Somerset Council	England – unitary authorities	15.825 (7)	The Healthy Workplace Awards	[82]	North Somerset Council	Local Government	NA	Local	No restrictions	General health	Free training & workshops, support for adopting healthy lifestyles & framework	Bronze, Silver & Gold
27. North Yorkshire Council	England – unitary authorities	14.762 (7)	Workplace Wellbeing Award North Yorkshire	[83]	North Yorkshire Council	Local Government	NA	Local	No restrictions	General health	Information & award	Bronze, Silver & Gold
28. Peterborough City Council	England – unitary authorities	27.821 (2)	Healthy Workplace Service	[84]	Peterborough City Council in partnership with Cambridgeshire County Council	Local Government	NA	Local	No restrictions	General health improvements including smoking cessation, weight management, physical activity & health checks	Face to face, virtual & telephone appointments to provide support & services	Not specified
29. Plymouth City Council	England – unitary authorities	26.619 (2)	The Wellbeing at Work Award/Wellbeing Training	[85]	Wellbeing at Work, a part of Livewell Southwest Commissioned by Plymouth City Council	Local Government	NA	Local	No restrictions	General health including healthy eating, alcohol awareness, mental health	Free training & workshops, support for adopting healthy lifestyles & framework	Bronze, Silver & Gold
30. Southend-on-Sea Borough Council	England – unitary authorities	22.375 (4)	Workplace Wellbeing	[86]	Southend-on-Sea Borough Council/Everyone Health	Local Government	NA	Local	No restrictions	General health	Free training & workshops, support for adopting healthy lifestyles & framework	Not specified
31. South Gloucestershire Council	England – unitary authorities	11.660 (9)	Workplace wellbeing	[87]	South Gloucestershire Council	Local Government	NA	Local	No restrictions	General health	Free training & resources	Not specified
32. Swindon Borough Council	England – unitary authorities	18.622 (5)	Workplace Health & Wellbeing	[88]	Swindon Borough Council	Local Government	NA	Local	No restrictions	General health, including physical & mental health, smoking cessation, alcohol awareness. There are specific areas, including dementia, diabetes, sexual health	Education classes, courses, support & online advices	Not specified
33. Barnsley Borough Council	England – Metropolitan district	29.933 (2)	Be Well @ Work awards	[89]	Barnsley Borough Council	Local Government	2019	Local	No restrictions	General health & wellbeing, including mental health support	Offers support through workplace visits, health & wellbeing surveys & advice tailored to business needs	Bronze, Silver & Gold
34. Coventry City Council	England – Metropolitan district	25.613 (3)	Thrive at Work Wellbeing Award	[90]	Coventry City Council	Local Government	NA	Local	No restrictions	General health & wellbeing, including mental health, MSK support & lifestyle	one-to-one telephone or virtual appointments, talks & workshops	Bronze, Silver & Gold
35. Doncaster Borough Council	England – Metropolitan district	30.289 (2)	Be Well @ Work	[91]	Doncaster Borough Council	Local Government	NA	Local	No restrictions	General health & wellbeing, including mental health support	Offers support through workplace visits, health & wellbeing surveys & advice tailored to business needs	Bronze, Silver & Gold
36. Newcastle Upon Tyne City Council	England – Metropolitan district	29.790 (2)	The Better Health at Work Award (BHAWA)	[92]	Newcastle Upon Tyne City Council	Local Government	2009	Local	No restrictions	General health	Increased access to health information & interventions	Bronze, Silver, Gold, Continuing Excellence, Continuing Excellence Plus & Maintaining Excellence
37. Oldham Borough Council	England – Metropolitan district	33.155 (1)	Workforce Health & Wellbeing Award	[93]	Oldham Borough Council	Local Government	2008	Local	No restrictions	Health & wellbeing	Award	Not specified
38. South Tyneside Borough Council	England – Metropolitan district	31.509 (1)	The Better Health at Work Award (BHAWA)	[94]	South Tyneside Borough Council	Local Government	2009	Local	No restrictions	General health	Increased access to health information & interventions	Bronze, Silver, Gold, Continuing Excellence, Continuing Excellence Plus & Maintaining Excellence
39. Salford City Council	England – Metropolitan district	34.210 (1)	Workplace health programme	[95]	Salford City Council has & Public Health England	Local Government	NA	Local	No restrictions	General health	Health & wellbeing needs assessment, health checks, workshops, wellbeing champions & trainings	Not specified
40. Sunderland City Council	England – Metropolitan district	30.586 (2)	The Better Health at Work award (BHAWA)	[96]	Sunderland City Council	Local Government	2009	Local	No restrictions	General health	Increased access to health information & interventions	Bronze, Silver, Gold, Continuing Excellence, Continuing Excellence Plus & Maintaining Excellence
41. Wakefield City Council	England – Metropolitan district	27.306 (2)	Wakefield Workplace Health & Wellbeing Charter	[97]	Wakefield City Council	Local Government	NA	Local	No restrictions	General health including physical (healthy eating, smoking cessation) & mental health	Not specified	Not specified
42. Wigan Borough Council	England – Metropolitan district	25.713 (3)	Workplace health	[98]	Wigan Borough Council	Local Government	NA	Local	No restrictions	General health including physical health checks & mental wellbeing	Not specified	Not specified
43. Richmond upon Thames	England – London boroughs	9.425 (10)	Local Healthy Workplace Award	[99]	London Borough of Richmond upon Thames	Local Government	NA	Local	No restrictions	General health. Support for wellbeing, mental health & wellbeing & healthy lifestyle promotion	Not specified	Not specified
44. Wandsworth	England – London boroughs	16.611 (6)	Local Healthy Workplace Award	[99]	Wandsworth Borough Council	Local Government	NA	Local	No restrictions	General health. Support for wellbeing, mental health & wellbeing & healthy lifestyle promotion	Not specified	Not specified
45. Cambridgeshire County Council	England—County	13.858 (8)	Healthy workplaces	[84]	Cambridgeshire & Peterborough	Local Government	NA	Local	No restrictions	General health	provision of resources & support to improve workplace health, NHS health checks, Mental Health first aid training	Not specified
46. Derbyshire County Council	England—County	18.392 (5)	Healthy Workplaces Derbyshire	[100]	Derbyshire County Council	County government	NA	Local	No restrictions	General health	Assessments, provision of resources & support to improve workplace health	Not specified
47. East Sussex County Council	England—County	19.770 (5)	East Sussex Wellbeing at Work	[101]	East Sussex County Council	County government	NA	Regional	All organisations excluding Brighton & Hove	General health	Health resources, training, events, signposting service & award	Commitment, Bronze, Silver & Gold
48. Essex County Council	England—County	17.016 (6)	Essex Working Well	[102]	Essex County Council & Maldon District Council	County government	NA	Regional	Those working in Thurrock & Southend-On-Sea are excluded	General health	Meetings, resources, trainings, development programme & e-newsletter	Level 1 (five actions), level 2 (additional six actions) & level 3 (further 10 actions)
49. Gloucestershire County Council	England—County	14.932 (7)	The Healthy Workplaces Gloucestershire	[60]	Gloucestershire County Council	County government	2019	Regional	No restrictions	General health	Assessments, provision of resources & support to improve workplace health	Foundation & Enhanced levels
50. Hampshire County Council	England—County	12.693 (8)	Workplace Wellbeing	[103]	Hampshire County Council	County government	NA	Regional	No restrictions	Mental health	Toolkits, actions plans, resources & support	Not specified
51. Hertfordshire County Council	England—County	18.89 (5)	Hertfordshire Healthy Workplace	[63]	Hertfordshire County Council	County government	2014	Regional	SMEs from the private & voluntary sector	General health	Provision of resources & training & support to develop workplace health champions	Level 1 (Foundation), level 2 (workplace health holistic view) & level 3 (local community health promotion)
52. Leicestershire County Council	England—County	12.330 (8)	Healthy workplaces	[104]	Leicestershire County Council	County government	2023	regional	No restrictions	General health	Offers free health & wellbeing services, resources, training & support for healthy lifestyle changes, workplace health needs assessment	Engaged (on sign-up), Committed (> 1 year), Empowered (> 2 years)
53. Norfolk County Council	England—County	21.183 (4)	Thriving Workplaces	[105]	Norfolk County Council	County government	2017	Regional	No restrictions	General health	Provision of resources & training & support to develop workplace health champions	Not specified
54. Nottinghamshire County Council	England—County	18.999 (5)	Wellbeing@Work	[106]	Nottinghamshire County Council	County government	NA	Regional	No restrictions	General health	Increased access to health information & interventions	Bronze, silver, Gold, Platinum, Maintenance
55. Staffordshire County Council	England—County	16.567 (6)	Workplace Health Staffordshire	[64]	Staffordshire County Council	County government	NA	Regional	No restrictions	General health	Awards programme & resources	Not specified
56. Surrey County Council	England—County	10.087 (10)	Workplace wellbeing	[107]	Surrey County council	County government	NA	Regional	No restrictions	General health	Provision of resources & training & support to develop workplace health champions	Not specified
57. Warwickshire County Council	England—County	15.640 (7)	The Workplace Wellbeing Forum	[108]	Warwickshire County Council & Coventry City Council	County government	2022	Regional	No restrictions	General health	A forum for local employers to meet, access resources & share best practice to help them implement or develop improved health & wellbeing support for their employees	Not specified
58. West Sussex County Council	England—County	14.429 (7)	Workplace health	[109]	West Sussex County Council	County government	NA	Regional	No restrictions	General health	Free training & workshops, face to face support for adopting healthy lifestyles & framework	Not specified
59. Worcestershire County Council	England—County	18.089 (6)	Work Well Live Better/Worcestershire Works Well	[110]	Worcestershire County Council	County government	2011	Regional	No restrictions	Mental health & wellbeing	Increased access to health information & interventions	Not specified
60. Dundee City Council	Scotland	Not applicable	Healthy Working Lives	[111]	Worcestershire County Council	County government	NA	Regional	< 250 employees	General health	offer training & information & awareness sessions for local companies who want to get involved	bronze, silver & gold
61. Ards & North Down Borough Council	North Ireland	Not applicable	Mind Body Business	[112]	Ards & North Down Borough Council	Local government	NA	Regional	No restrictions	General health	Increased access to health information & interventions	Not specified

### Synthesis of results

Our objective was to identify and characterise the provision of WHISPAs across the UK.

#### Identification and distribution

**Fig. 2:**
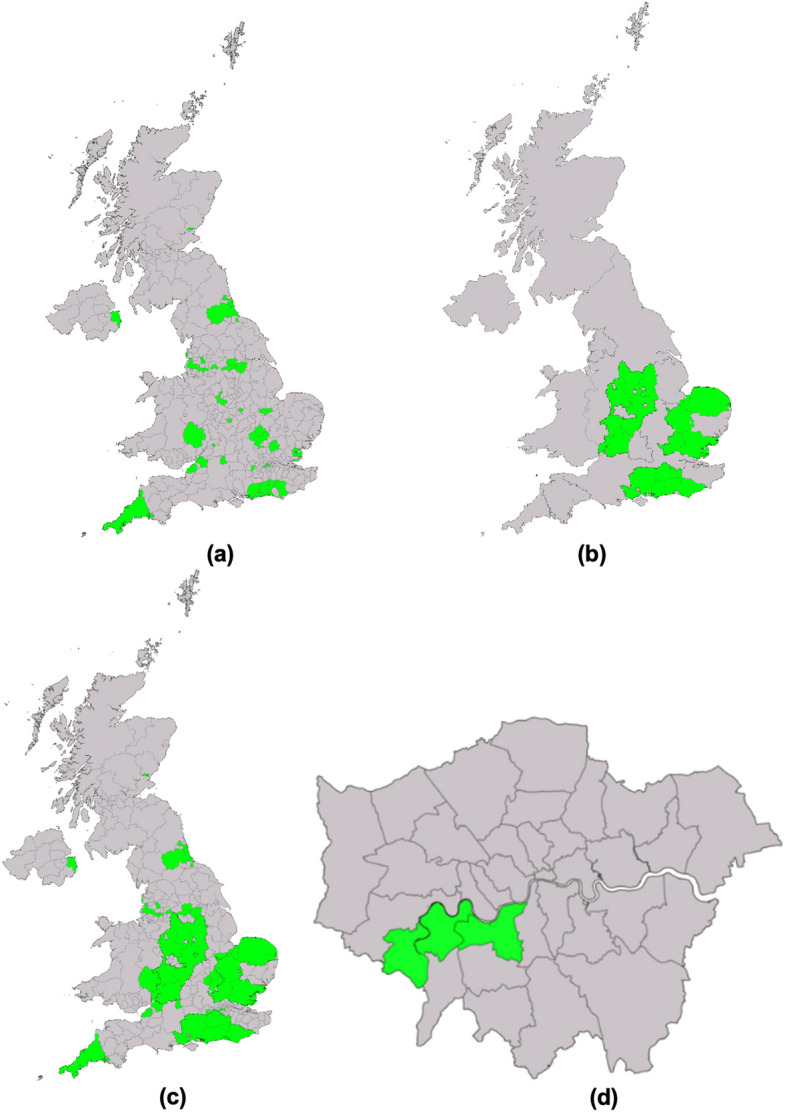
Geographical distribution of 61 WHISPAs across the UK presented as a map of (**a**) Local Authority Districts, (**b**) Counties, (**c**) Combined (or unitary) Local Authorities, and (d) London boroughs and the City of London

There were 61 WHISPAs identified in local councils across the UK. The visualisation of WHISPAs across the UK presented in Fig. 2 highlights a higher concentration in urban areas and regional hubs. Some rural regions exhibited limited or no offerings, suggesting a disparity in access based on geographical location.

WHISPAs were unevenly distributed across the UK, with 59 (96%) interspersed in England, one in Scotland, and one in Northern Ireland, whereas no eligible WHISPAs were identified in Wales. Whilst Healthy Working Wales (a national programme) was active and free for workplaces, it was excluded because it was offered by Public Health Wales and not the local government.

The initiatives within England were distributed across various administrative divisions, suggesting a higher density in urban areas and regional hubs, while some rural regions exhibited limited or no offerings, indicating geographical disparities in access (13 were in non-metropolitan districts, 19 were in unitary authorities, 10 were in metropolitan districts, 15 counties and two London boroughs). For some areas, there could be the same WHISPA offered at the district level (lower tier) and the county level (upper tier) or two different WHISPAs offered at these two levels.

In Northern Ireland, the Work Well, Live Well initiative was offered by the Public Health Agency (PHA) as part of the Health and Social Care (HSC) but not the local government [77]. Therefore, this initiative was excluded also despite meeting all the other inclusion criteria including being free to access.

#### Administration, implementation and provider type

The administering bodies varied, with programmes delivered directly by local government (*n* = 28, 45.9%), county government (*n* = 17, 27.9%), both local and county government (*n* = 12, 19.7%) or through partnerships with healthcare providers, non-governmental organisations or governmental bodies rather than local authorities (*n* = 4, 6.5%). As these initiatives could be accessed free of charge to employees, many were designed to be inclusive, offering language support and accessibility for individuals with disabilities. The level of documentation and promotion of these initiatives also varied, with some well-documented through local government channels and others relying primarily on word-of-mouth or internal communications.

#### IMD Score

The IMD scores for the WHISPAs itemised in Table 1 highlight the socio-economic diversity of the regions they serve. Scores ranged from highly deprived areas, such as Blackpool with a score of 45.039 in the 1 st decile, to less deprived areas, like Bracknell Forest with a score of 10.241 in the 10 th decile. Most WHISPAs were concentrated in areas with moderate to high levels of deprivation, typically falling within the 5 th to 7 th deciles. Specifically, 20 WHISPAs (33%) were in the 5 th decile, 12 (20%) in the 6 th decile, and 9 (15%) in the 7 th decile. This indicates a focus on providing workplace health initiatives in regions where socio-economic challenges are more prevalent, aiming to address and mitigate workplace health disparities in these areas.

#### Year established

The years of establishment available for 22 WHISPAs (36%) were identified, with some programmes dating back to 2008 whereas others were initiated as recently as 2024. This timeline reflects the growing recognition over the years of the importance of workplace health and the evolving strategies to address health concerns within different workforce populations.

#### Coverage

The coverage of these initiatives was primarily localised, targeting the workplaces within the district or county.

#### Eligibility for workplaces

Most WHISPAs (*n* = 56, 91.8%) had inclusive eligibility criteria, with no restrictions on workplace participation. This inclusivity allows a wide range of workplaces, including SMEs from the private and voluntary sectors, to benefit from these initiatives.

#### Primary health focus

All 61 initiatives (100%) prioritised general health and/or mental wellbeing, reflecting current health trends and workplace challenges. In addition to the primary focus we reported some specific conditions such as MSK issues, smoking cessation, anxiety and healthy eating.

#### Scheme type

The types of schemes provided by WHISPAs varied widely, from one-to-one consultations and workshops to comprehensive health assessments and resource and materials provision.

#### Accreditation

Accreditation levels also varied among the WHISPAs, with many initiatives (*n* = 24; 39%) offering formal recognition levels like Bronze, Silver, Gold and even Platinum. The names of the levels, number of levels and requirements to be promoted from one level to another differ between WHISPAs. These accreditation schemes recognised progress and incentivised continuous improvement in workplace health practices, providing benchmarks that encourage workplaces to achieve higher standards of health and wellbeing. Some councils developed structured programmes offering tiered levels of engagement, such as foundational, enhanced and specialised interventions, designed to cater to the varying needs and capacities of workplaces.

## Discussion

### Summary of main findings

Our scoping review of Workplace Health and wellbeing initiatives that are Free at the Point of Access (WHISPAs) across the UK identified a total of 61 free and currently active programmes, demonstrating a considerable effort across Local Authorities (LA) to address workplace health. These initiatives spanned a broad spectrum of health, primarily targeting general health and mental wellbeing. The findings also highlighted a potential significant disparity in geographic distribution with a concentration of initiatives in certain regions in England compared to the rest of the UK.

### Interpretation

The clustering of WHISPAs primarily in certain areas highlights a notable disparity in the availability and accessibility of such programmes. This distribution reflects the concentration of resources and organisational infrastructure which tend to have better access to funding, expertise and networks necessary to implement and sustain health initiatives. Conversely, despite some areas (e.g., industrial areas) having higher employment, they may face challenges such as limited funding, fewer health professionals and logistical complexities that hinder the establishment and maintenance of similar programmes [113].

The focus of the identified WHISPAs on general health and mental wellbeing mirrors the increasing global awareness of the critical impact of mental health on overall life satisfaction and productivity. Mental health issues which are often exacerbated by work-related stress can significantly affect employee performance and are increasingly recognised as a key area for intervention by employers [114]. This alignment with global trends indicates a responsive adaptation of workplace initiatives to current health priorities, yet it also raises questions about the breadth and depth of these initiatives'focus. For example, are physical health issues, particularly those exacerbated by workplace environments such as ergonomic problems or occupational diseases, being adequately addressed?

Additionally, the high visibility of mental health and wellbeing programmes within these initiatives could reflect an evolving understanding of workplace health as a holistic concept that includes emotional and social wellbeing, not just physical health. This shift is consistent with contemporary health promotion models that emphasise the importance of a supportive environment as essential for fostering healthy lifestyle choices among employees [115]. The findings also suggest a potential oversight in integrating WHISPAs into broader public health strategies. While these programmes are crucial, their impact may be limited if they are not effectively integrated with local and national health policies. Effective integration could enhance the reach and efficacy of these programmes, ensuring they are part of a coordinated effort to improve health outcomes across all population sectors. This interpretation of our results highlights the necessity for a strategic approach to address the inequities in the distribution of WHISPAs and to ensure that the focus on mental health does not overshadow the need for comprehensive health strategies that include physical health aspects [116].

The observed regional disparities in WHISPA provision highlight the need for more coordinated strategic planning at both the local and national levels. Integrating workplace health initiatives into existing local frameworks, such as Joint Strategic Needs Assessments and Local Industrial Strategies [42], could help ensure that resources are targeted to areas of greatest need and that programmes are sustained over time [117, 118]. Furthermore, the development of a national accreditation framework could increase the visibility and uptake of effective workplace health programmes, supporting LAs in their efforts to address both physical and mental health in the workforce [53].

### Strengths and limitations

To our knowledge, this is the first national study on WHISPAs in the UK. This landscape analysis resulting form this research could help raise awareness about contemporary initiatives and inform research and policy directions to optimise the use of WHISPAs to improve workplace health and wellbeing.

The principal limitation of this study was concerned with our primary reliance on publicly available information, supplemented by direct council responses and information provided following a Freedom of Information request which is a laborious process. For these reasons we acknowledge we may not have fully captured all active initiatives, particularly in areas with limited online documentation or non-responsiveness. Another limitation is that the vast majority of initiatives identified were from England, limiting direct comparability across the four nations. A third limitation is concerned with the lack of other contextual information including the number of employees or the total population served by each local authority. As these data were not consistently available across all nations they was not systematically included.The fourth limitation is that we the deprivation analysis was restricted to English local authorities due to the lack of harmonised deprivation indices across the UK. For this reason, findings related to deprivation and WHISPA provision are specific to England.

We also acknowledge that we did not assess the quality, implementation fidelity, or effectiveness of the identified programmes. Finally, our findings regarding regional disparities and gaps in provision should be interpreted with caution as our data cannot be defined as representative of the UK we cannot argue that this is certainly a significant disparity. Future research should address these limitations by incorporating more comprehensive contextual data, harmonising deprivation measures, involving local collaborators across all UK nations, and evaluating the reach and impact of workplace health initiatives.

### Implications for practice

The findings of our scoping review point to several key implications for practice that could enhance the effectiveness and reach of WHISPAs across different regions and sectors (Table 3). To address the disparity observed in the distribution of WHISPAs, practitioners should consider partnerships that can leverage existing infrastructure to facilitate the delivery of health programmes including with local community organisations and health providers. Mobile health units, telehealth services and local health fairs are also potential methods to extend the reach of WHISPAs to these underserved areas. However, because many SMEs also lack the resources to implement comprehensive health programmes, practitioners should develop scalable and flexible health promotion models that are financially and logistically feasible for smaller organisations. Offering tiered levels of engagement and using digital platforms to deliver health education and interventions can make WHISPAs more accessible to SMEs.

**Table 3:** Key issues in implementing effective workplace health and wellbeing initiatives that are Free at the point of access

1. Funding and resources, including securing sustained funding to cover the costs of programmes, including materials, health professionals and program maintenance.
2. Employee engagement to encourage consistent participation among employees, especially in diverse workforces with varying health needs and interests.
3. Data privacy and trust when managing personal health data sensitively to maintain trust, especially concerning mental health and other sensitive issues.
4. Measuring effectiveness to help demonstrate the social and financial return on investment potential of health and wellbeing initiatives, including indirect benefits such as improved morale or reduced staff turnover.
5. Integration with work practices to ensure congruent integration of health and wellbeing programmes into the daily routines of employees without disrupting productivity.
6. Enhance accessibility so programmes can be accessed by all employees, including remote workers and those on different shifts.
7. Comprehensive approach in developing programmes that address both physical and mental health in a holistic manner.
8. Compliance with regulations including adhering to statutory health and safety regulations.
9. Cultural change to positively impact the prevailing organisational culture to increasingly prioritise health and wellbeing.

Given the prevailing focus on mental health in the identified WHISPAs and other studies, there is a clear need to promote these initiatives better. Employers should be encouraged to integrate mental health strategies into their overall health and safety policies including training for managers to recognise signs of mental distress, providing mental health first aid training and establishing clear pathways for support and counselling. It is also crucial that organisations enhance their communication strategies to overcome the barriers related to awareness and perceived irrelevance. This could involve regular workshops, newsletters and interactive sessions that not only inform but also engage employees in the design and implementation of health initiatives. Effective communication should highlight the direct benefits of participation, address common misconceptions and emphasise the personal and professional advantages of improved health.

The continuous evaluation of WHISPAs should also be standard practice. This will help ensure that they remain relevant and effective, whereas feedback mechanisms should be integrated into all initiatives, allowing for ongoing assessment and adaptation based on employee needs and health outcomes.

### Implications for research

Our study highlighted several areas where further research is needed to enhance understanding and improve the implementation and effectiveness of these initiatives. Future studies should focus on assessing the effectiveness of different types of WHISPAs, particularly through longitudinal research designs that measure both short-term and long-term health outcomes. This includes exploring the effect of the WHISPA on physical health metrics as well as mental health improvements and socio-economic outcomes such as productivity and employee retention.

Because WHISPAs with more quality assured assets could be considered to have higher credibility, a more comprehensive review could also include a content assessment of the documentation provided including formal reports, participant testimonials, evidence of regular reviews and updates. The transparency of program operations and the ease of access to information should also be considered since those initiatives that provide clear and accessible descriptions of their WHISPAs, including eligibility criteria, enrolment processes and points of contact, could also be assumed to have higher credibility. Finally, future research could evaluate WHISPAs against recognised best practices in workplace health promotion (e.g., the extent to which they integrated holistic health approaches, such as addressing both physical and mental health, adhering to privacy regulations and offering personalised health support as models of comprehensive health promotion efforts).

There is also a need to investigate which elements of program design and implementation are most effective in engaging employees and promoting sustained participation. This would include exploring the impact of program customisation based on demographic factors such as age, gender and occupation as well as organisational factors like company size and industry. Given the rise of digital health solutions, future research should also evaluate the effectiveness of technology-driven WHISPAs [119, 120], including the use of mobile apps, wearable devices and telehealth services within workplace settings to determine their impact on employee health and their scalability across different workplace environments. Future studies should aim to provide clear data on the social and financial return on the investment potential of these programmes, considering both direct healthcare costs and indirect costs related to productivity and absenteeism to drive evidence-based commissioning. Finally, more research into the cultural and ethical considerations surrounding WHISPAs is needed to ensure that these programmes are inclusive and respect participants'diverse backgrounds and privacy, including understanding how cultural norms influence the acceptance and effectiveness of health initiatives. Some of the challenges identified in this review may be rooted in the underlying business models and operational priorities chosen by employers, which can influence the adoption and success of workplace health and wellbeing initiatives. While evaluating the effectiveness and organisational context of these programmes was beyond the scope of this study, future research should examine how different business models and workplace cultures impact both the implementation and outcomes of WHISPAs.

## Conclusion

This scoping review identified 61 Workplace Health and wellbeing initiatives that are Free at the Point of Access (WHISPAs) with an uneven distribution across the UK. By addressing the identified gaps and leveraging the insights gained, stakeholders can enhance the design, implementation and promotion of WHISPAs by learning from best practice examples and, in this way, help promote a healthier and more productive workforce across both urban and rural areas.

## Data Availability

The protocol and data extraction tables can be made available upon request.

## Abbreviations

ARC: Applied Research Collaboration
FOI: Freedom of Information
FOSS: Free and Open-Source Software
IMD: Index of Multiple Deprivation
LA: Local Authority
LAD: Local Authority District
MSK: Musculoskeletal
NIHR: National Institute for Health and Care Research
ONS: Office for National Statistics
SME: Small or medium enterprise
UK: United Kingdom
WHISPA: Workplace Health and Wellbeing Initiatives that are Free at the Point of Access
